# Bone scintigraphy imaging and transthyretin-related (ATTR) cardiac amyloidosis: New tricks from an old tool?

**DOI:** 10.1007/s12350-022-03032-2

**Published:** 2022-07-22

**Authors:** Jessica M. Duran, Salvador Borges-Neto

**Affiliations:** 1 Division of Cardiology, Department of Medicine, Duke University School of Medicine, Durham, NC USA; 2 Division of Nuclear Medicine, Department of Radiology, Duke University School of Medicine, Durham, NC USA

Amyloidosis is a heterogenous group of diseases characterized by progressive organ dysfunction caused by deposition of misfolded proteins in various target organs including the heart and the nervous system. The two most common types of amyloidosis that affect the heart are immunoglobulin light chain cardiac amyloidosis (AL-CA) and transthyretin cardiac amyloidosis (ATTR-CA). ATTR-CA is an infiltrative cardiomyopathy that arises secondary to deposition of insoluble transthyretin amyloid fibrils in the extracellular space of the myocardium. The natural history of ATTR-CA can be variable, but if untreated is characterized by progressive heart failure, conduction system disease, arrhythmias, and ultimately death, which typically occurs only several years after diagnosis.^1^ ATTR-CA can be classified as hereditary (hATTR), in which an autosomal dominant pathogenic mutation is present in the *TTR* gene, or wild-type (wtATTR), an acquired disorder in which no mutation is identified. There are specific *TTR* mutations associated with the development of a polyneuropathy (PNP), while others are predominantly associated with a cardiomyopathy or a mixed phenotype.^2^

Historically considered a rare disease, cardiac amyloidosis is currently recognized as a significantly more prevalent clinical entity.^3,4^ Now in the era of available therapeutic agents^5–8^ that can slow or halt disease progression, the early identification of patients with ATTR-CA has become increasingly more important than ever. Technetium-labeled cardiac scintigraphy (^99m^Tc-PYP, ^99m^Tc-DPD, and ^99m^Tc-HMDP) plays a critical role in the non-invasive diagnostic pathway for patients with suspected ATTR-CA.^9^ Advances in bone scintigraphy coupled with increased awareness have led to an exponential increase in the number of patients diagnosed with ATTR-CA. Furthermore, no longer requiring an endomyocardial biopsy to make the diagnosis and instead in many cases being able to non-invasively diagnosis ATTR-CA in appropriately selected patients (myocardial uptake of radiotracer grade ≥2 and the absence of a clonal plasma cell process) has contributed to increasing diagnoses.^10,11^

In addition to providing information about possible cardiac involvement in patients with suspected ATTR-CA, DPD scintigraphy can also reveal extra cardiac uptake of tracer. Several prior studies have reported characteristic extra cardiac uptake in soft tissues in patients with ATTR amyloidosis. In a study by Hutt et al., a cohort of patients with suspected or proven cardiac amyloidosis underwent DPD scintigraphy.^12^ In contrast to patients with AL-CA, patients with ATTR-CA had both cardiac uptake as well as extensive soft tissue uptake in the shoulder, gluteal, and abdominal wall muscles. A later study by the same group reported higher soft tissue to femur ratio in relation to Perugini grade using DPD scintigraphy in patients with ATTR amyloidosis.^13^

In this issue of the *Journal of Nuclear Cardiology,* Wollenweber et al. reported the potential utility of increased [^99m^Tc]-3,3-Diphosphono-1,2-Propanodicarboxylic Acid (DPD**)** soft tissue uptake to serve as an imaging marker that identifies patients with ATTR-CA at a higher risk for having concomitant PNP.^14^ The authors of this study hypothesized that there would be higher soft tissue DPD uptake in ATTR-CA patients with PNP as compared to ATTR-CA patients without PNP. In this retrospective, single center study, a cohort of 50 patients with ATTR-CA (41 wild-type and 9 hereditary) who had undergone whole body DPD scintigraphy and a nerve conduction study (NCS) were evaluated. The study cohort was predominantly male (80%) with a mean age of 77.4 ± 8.2 years. The diagnosis of ATTR-CA was made on the basis of the absence of a monoclonal protein in the blood or urine^10^ and a Perugini^14^ grade 2 or 3 (36 patients), an endomyocardial biopsy (12 patients), or a positive DPD scan and genetic testing (2 patients). NCS results revealed that 50% of patients (19 wtATTR and 6 hATTR) had a sensorimotor PNP, 20% had a sensory PNP (9 wtATTR and 1 hATTR), and the remaining patients (13 wtATTR and 2 hATTR) did not have a PNP. The mean time interval between the bone scan and NCS was 210 ± 93 days. A subgroup of the study cohort (22 patients) also underwent quantitative SPECT/CT of the thorax directly after planar imaging. As SPECT/CT image acquisition was primarily performed over the thorax, the subcutaneous abdominal fat was not available for evaluation in all patients. Therefore, standardized uptake values in the subcutaneous fat tissue of the left axillary region were evaluated instead.

The results from the visual DPD bone scintigraphy analysis revealed that the majority of patients with wtATTR (97.6%) and hATTR (100%) had a Perugini grade of 2 or 3. Furthermore, whole body imaging analysis of the entire study cohort demonstrated a significantly higher Perugini score in patients with ATTR-CA and PNP as compared to patients with ATTR-CA and without PNP. Excluding patients with a history of diabetes mellitus from the analysis, further increased the significance of this finding between the group of patients with ATTR-CA with and without PNP. In patients with wtATTR and without a history of diabetes mellitus, there was also a statistically higher Perugini score observed in patients with PNP as compared to those without.

The analysis of quantitative DPD bone scintigraphy revealed that even after exclusion of patients with a history of diabetes mellitus, there was not a statistically significant difference between whole body DPD uptake normalized to the applied activity between ATTR-CA patients with and without PNP. Furthermore, region of interest (ROI) analysis revealed that skull uptake was significantly decreased in ATTR-CA patients with as compared to without PNP. This finding was also observed in the subgroup of patients with wtATTR.

Regarding the thoracic SPECT/CT group analysis, there was significantly increased DPD uptake in the left axillary region subcutaneous fat in ATTR-CA patients with as compared to without PNP. Soft tissue DPD uptake was still significantly increased in ATTR-CA patients with as compared to without PNP after exclusion of patients with diabetes. In a subgroup analysis of patients with wtATTR, there was not a statistically significant difference in SUV_peak_ in the left axillary region subcutaneous fat between patients with and without PNP irrespective of history of diabetes mellitus.

Although relatively small and retrospective in nature, this study does have merit. Not only does this study contribute to the current ATTR-CA knowledge base, but it also expands the potential diagnostic utility of using DPD scintigraphy in patients with suspected ATTR-CA. The observation that patients with ATTR-CA with PNP have increased soft tissue DPD uptake is in line with prior studies that have utilized quantitative bone-avid radiotracer imaging and have reported a correlation between tracer uptake and cardiac amyloid burden and phenotype.^15–18^ The use of quantitative bone-avid cardiac scintigraphy to quantify amyloid burden in ATTR-CA is currently an area of great interest and holds the promise of aiding with early disease diagnosis, risk stratification, prognostication, assessment of treatment response, and potentially improvement in patient outcomes.^19^ The findings from this current study suggest that a higher Perugini score and increased DPD soft tissue uptake in subcutaneous fat could potentially be a clinically useful diagnostic imaging marker to identify patients with ATTR-CA and PNP. If so, this observation would further expand the diagnostic utility of DPD scintigraphy to identify not only patients with ATTR-CA, but also to potentially identify patients with PNP. Furthermore, this observation underscores a potential added benefit of utilizing DPD over PYP scintigraphy in being able to quantify extracardiac radiotracer uptake in the diagnostic evaluation of patients with suspected ATTR-CA.

It is however worth mentioning that this study does have several limitations. As previously mentioned, the primary limitation is its small sample size and retrospective nature. Furthermore, the association of a higher Perugini score with PNP observed in this study cannot rule out increased DPD uptake secondary to more advanced disease. Lastly, the extended time interval between the bone scan and NCS, lack of details surrounding the onset of PNP, and not excluding other causes of neuropathy with the exception of diabetes mellitus, represent additional limitations of the study.

In summary, ATTR-CA is an increasingly recognized cause of cardiomyopathy and/or PNP. Given the progressive nature of untreated ATTR-CA and the availability of disease modifying therapies, identifying patients early in their disease course remains of critical importance. The use of DPD bone scintigraphy plays an important role in the non-invasive diagnostic evaluation of a patient with suspected ATTR-CA. Furthermore, the findings from this study suggest that increased soft tissue DPD uptake could be used as a potential diagnostic imaging marker to identify patients with ATTR-CA with an increased risk of PNP. Future prospective, randomized control studies are needed to confirm these observations on a larger scale and further clarify the clinical significance of increased soft tissue DPD uptake in patients with ATTR-CA.
